# *Aralia continentalis* Root Enhances Non-Rapid Eye Movement Sleep by Activating GABA_A_ Receptors

**DOI:** 10.3390/nu15245020

**Published:** 2023-12-06

**Authors:** Minseok Yoon, Dong Wook Lim, Jonghoon Jung, Young Sung Jung, Changho Lee, Min Young Um

**Affiliations:** 1 Division of Functional Food Research, Korea Food Research Institute, Wanju-gun 55365, Republic of Korea; msyoon@kfri.re.kr (M.Y.); dwlim@kfri.re.kr (D.W.L.); jonghoon@kfri.re.kr (J.J.); j.youngsung@kfri.re.kr (Y.S.J.); 2 Division of Food Biotechnology, University of Science & Technology, Daejeon 34113, Republic of Korea

**Keywords:** *Aralia continentalis*, sleep, hypnotic, GABA_A_ receptor

## Abstract

*Aralia continentalis* exhibits various biological activities; however, their sleep-promoting effects have not been previously reported. In this study, we evaluated the hypnotic effects and sleep–wake profiles of *A. continentalis* root (KS-126) using a pentobarbital-induced sleep-acceleration test and polysomnographic recordings. Additionally, we investigated the molecular mechanism of KS-126 through patch-clamp electrophysiology. Our polysomnographic recordings revealed that KS-126 not only accelerated the onset of non-rapid eye movement sleep (NREMS) but also extends its duration. Considering the temporal dynamics of the sleep–wake stages, during the initial and subsequent periods KS-126 extended NREMS duration and decreased wakefulness, thereby enhancing sleep-promoting effects. Furthermore, the assessment of sleep quality via analysis of electroencephalogram power density indicated that KS-126 did not significantly alter sleep intensity. Finally, we found that KS-126 enhanced GABA_A_ receptor-mediated synaptic responses in primary hippocampal neurons, leading to an increase in the percentage of the GABA current. This effect was not affected by the selective benzodiazepine receptor antagonist flumazenil, but was entirely inhibited by the GABA_A_ receptor antagonist bicuculline. In conclusion, KS-126 extends the duration of NREMS without altering its intensity by prolonging GABAergic synaptic transmission, which modulates GABA_A_ receptor function.

## 1. Introduction

Sleep is a fundamental part of our lives, constituting nearly one-third of our time, and is closely related to health [1]. Approximately 10% of the world’s population experience severe insomnia and more than one-third report insufficient daily sleep [2]. Insomnia negatively affects physical health, such as cardiovascular and immune function, as well as mental health, including memory and mood [3]. Benzodiazepines (BDZ) and non-BDZ are the two classes of insomnia medications that act on γ-aminobutyric acid (GABA)_A_ receptors. Recently, a meta-analysis indicated that both BDZ and non-BDZ exhibit similar effects in the short-term treatment of sleep disorders [4]. However, these hypnotic agents are not recommended for long-term use due to their potential side effects, including daytime drowsiness, cognitive impairment, and risk of dependence [5].

Recently, there has been increasing interest in the use of alternative therapies, including natural products, to treat insomnia [6]. Several natural products such as phlorotannins, L-tryptophan, 5-hydroxytryptophan, Valeriana officinalis, and *Hypericum perforatum* can improve sleep without severe side effects [7]. Saponins from *Ziziphus jujube* seeds exert hypnotic effects on pentobarbital-induced sleep in mice models [8]. Schisantherin A from *Schisandra chinensis* increases the number of sleeping periods and sleep time in mice by regulating GABA_A_ expression in mice and rats [9]. Marine polyphenol phlorotannins derived from *Ecklonia cava* demonstrate sedative-hypnotic effects in mice by acting through the GABA_A_-BDZ receptor [10,11]. These reports suggest that several natural products possess various hypnotic properties. Thus, it is essential to scientifically investigate the hypnotic impacts of diverse natural compounds.

*Aralia continentalis*, found in Korea, China, and Japan, is a well-known edible medicinal herb used in traditional prescriptions for the treatment of rheumatism, neuralgia, lumbago, and inflammation [12]. The roots of *A. continentalis* have been reported to have analgesic [13], neuroprotective [14], anti-osteoarthritic [15], and anti-apoptotic [16] activities. Continentalic and kaurenoic acids are the primary diterpenoids found in the roots of *A. continentalis*. Several of these diterpenoids have been reported to possess various beneficial activities, including anti-inflammatory [17,18], anti-cancer [19], and wound-healing activity [20], and anti-convulsant [21] properties. However, the sleep-promoting effects of *A. continentalis*, including its active compounds, have not been investigated yet. In the present study, we aimed to investigate the hypnotic effects of *A. continentalis* root extract (KS-126) on pentobarbital-induced sleep in mice. Additionally, we examined potential changes in sleep architecture and profiles. Furthermore, we assessed the involvement of GABAergic mechanisms in these effects.

## 2. Materials and Methods

### 2.1. Chemicals and Reagents

Zolpidem (ZPD) and pentobarbital were obtained from the Ministry of Food and Drug Safety (Cheongju, Republic of Korea) and Hanlim Pharmaceutical Co., Ltd. (Seoul, Republic of Korea), respectively. Flumazenil and bicuculline were purchased from Sigma-Aldrich (St. Louis, MO, USA). All other reagents were of the highest commercially available grade. Analytical-grade dimethyl sulfoxide (DMSO) was purchased from Sigma-Aldrich Co., LLC (St. Louis, MO, USA). Continentalic acid (CA) and kaurenoic acid (KA) were purchased from Chemfaces (Wuhan, China). High-performance liquid chromatography (HPLC)-grade water, acetonitrile, methanol, and chloroform were purchased from Thermo Fisher Scientific (Waltham, MA, USA). Polytetrafluoroethylene (PTFE) filters were purchased from the Pall Corporation (0.45 μm; Port Washington, NY, USA).

### 2.2. Preparation of KS-126

The dried roots of *A. continentalis* were obtained from the medicinal plant market in Jecheon, Republic of Korea, and were taxonomically confirmed by Dr. Sang Yoon Choi of the Korea Food Research Institute. They were extracted using 70% ethanol solution (*v*/*v*) at 80 °C for 3 h using a reflux cooling apparatus. The extract was then lyophilized to yield the final extract (KS-126, which was 19.27% (*w*/*w*).

### 2.3. High-Performance Liquid Chromatography Analysis

Analytical grade CA and KA were prepared by dissolving them in 100 μL of DMSO and then in methanol/chloroform (1:1, *v*/*v*) at 10 mg/mL. Standard working solutions were prepared by diluting each stock solution with absolute methanol. Stock solutions were stored in the refrigerator at −20 °C in amber glass vials (Waters Corp., Milford, MA, USA). The KS-126 was weighed at 15 mg and dissolved in 100 μL of DMSO and then methanol/chloroform (1:1, *v*/*v*) at 15 mg/2 mL. The analytical samples were diluted in absolute methanol to 200 ppm and were filtered through a 0.45 μm PTFE filter (Pall Corp.). The standards and samples were analyzed using a reverse-phase HPLC system (PU-2080 Plus; JASCO, Tokyo, Japan) with an ultraviolet detector (PU-2075 Plus; JASCO). A YMC Hydrosphere C18 column (4.6 mm × 250 mm, 3.0 μm; YMC, Tokyo, Japan) was used for chromatographic separation. The mobile phase consisted of a mixture of 80% (*v*/*v*) acetonitrile in water. Each injection (10 µL) was subjected to an isocratic elution with a constant flow rate of 1.0 mL/min, lasting for 25 min. The column temperature was held steady at 30 °C. The levels of KA and CA were assessed at a wavelength of 205 nm.

### 2.4. Animals

C57BL/6N (11 weeks, male) and ICR (4 weeks, male) mice were obtained from Kotech Co., Ltd. (Pyeongtaek, Republic of Korea). They underwent a one-week acclimation period in the cage before being used in the experiments. The animals were housed in a controlled environment with a temperature maintained at 23 ± 1 °C, relative humidity at 55 ± 5%, and a 12 h light-dark cycle (lights on from 07:00), while the light intensity was set to 3000 Lux. All procedures involving animals were carried out in compliance with the regulations and guidelines set by the Institutional Animal Care and Use Committee of the Korea Food Research Institute, with permission numbers KFRI-M-22015 and KFRI-M-22051. The experiments followed the ARRIVE guidelines 2.0.

### 2.5. Pentobarbital-Induced Sleep Accelerated Test

In most previous studies, the animals employed in the pentobarbital-induced sleep accelerated test were ICR mice [22,23,24,25]. Thus, in this study, ICR mice were chosen for the pentobarbital-induced sleep accelerated test, following our previously established reports by Um et al. [26]. All experiments were conducted between 13:00 and 17:00, and the ICR mice were used after a 24 h fasting period before the experiment. Prior to administration, all samples were dissolved in sterile saline containing 5% Tween 80 and subsequently given to the mice orally (p.o.) using an animal-feeding needle (oral Zonde). The dosages of KS-126 and ZPD were selected prior to our experiment based on the relevant literature [27,28]. ZPD was used as the positive control drug. Fifty mice were randomly assigned to five groups, each containing ten mice: (1) the control group; (2) the ZPD 10 mg/kg-treated group; (3) the KS-126 250 mg/kg-treated group; (4) the KS-126 500 mg/kg-treated group; and (5) the KS-126 1000 mg/kg-treated group. After oral administration of the sample to mice, pentobarbital (45 mg/kg) was intraperitoneally (i.p.) administered 45 min later. Sleep duration in mice administered pentobarbital was determined by measuring the time difference between the loss and recovery of the righting reflex. Prior to administration, all samples were dissolved in sterile saline containing 5% Tween 80 and subsequently given to the mice orally (p.o.).

### 2.6. EEG Surgery, Recording and Data Analysis

A head mount from Pinnacle Technology Inc. (Lawrence, KS, USA) for polysomnographic recordings was chronically implanted into the skulls of the C57BL/6N mice. Implantation was performed with the animals under pentobarbital anesthesia (50 mg/kg, i.p.). C57BL/6N mice were also chosen for recording and data analysis through EEG surgery, following established procedures and methods in previous reports [29,30]. To enable electroencephalogram (EEG) measurements, the head mount was precisely placed with its front edge 3.0 mm anterior to the bregma. Subsequently, four electrode screws were carefully placed into the predrilled holes in the skull, and two EMG wires were inserted into the nuchal muscles. Finally, the head mount was securely fixed using dental cement. Surgically treated mice were allowed a one-week recovery period in separate cages to ensure sufficient rest and recovery. All samples were dissolved in sterile saline containing 5% Tween 80 and subsequently given to the mice orally (p.o.) using an animal-feeding needle (oral Zonde). In the sleep architecture analysis, the sample dosage was selected following the result of a pentobarbital-induced sleep accelerated test. Twenty-four mice were randomly divided into three groups, each with eight mice: (1) the control group; (2) the ZPD 10 mg/kg-treated group; and (3) the KS-126 500 mg/kg-treated group. Before the experiment, the mice underwent a period of adaptation to the recording conditions for four days to reduce the influence of first-night effects. Polygraphic recordings were conducted using a sleep ring specifically designed to enable the free movement of the mice during the recording. The recordings were obtained using a data acquisition system from Pinnacle Technology, Inc. (PAL-8200, Lawrence, KS, USA). Polygraphic signals were stored at a sampling rate of 200 Hz and amplified (100×). Additionally, the EEG was low-pass filtered at 25 Hz and the EMG was low-pass filtered at 100 Hz. Both the baseline and experimental days were monitored for 48 h to observe the sleep–wake states. The baseline for each animal was recorded from 17:00 and continued for 24 h. These initial recordings acted as control measures for the corresponding animals, offering a reference for comparison throughout the course of the experimental day. Vigilance states, including wakefulness (Wake), rapid eye movement sleep (REMS), and non-REM sleep (NREMS), were automatically classified into 10 s epochs using SleepSign ver. 3.0 (Kissei Comtec, Tokyo, Japan). Finally, the sleep–wake stages were visually inspected and any necessary adjustments were made. The delta activity of the NREMS was firstly aggregated and subsequently standardized, represented as a percentage relative to the average delta activity during NREMS.

### 2.7. Patch-Clamp Electrophysiology

#### 2.7.1. Primary Neuron Culture

Primary neuron cultures were prepared from postnatal day 0 of ICR mice as described [31]. Briefly, the hippocampi of mouse pups were dissected to remove any attached meninges, then finely minced, and transformed into a single-cell suspension through trituration. Isolated cells were plated on glass coverslips coated with poly L-ornithine hydrobromide (Sigma-Aldrich, St. Louis, MO, USA) in 12-well plates. Cells were grown in Neurobasal A medium (Thermo Fisher Scientific, CA, USA) supplemented with 2% B27, 2 mM L-glutamine, and 0.5× penicillin-streptomycin. The cultured neurons were incubated in a humidified incubator at 37 °C with 5% CO_2_. Every three days, one-third of the culture medium was replaced with fresh medium. During medium replacement, 1 μM of Cytosine *β*-D-arabinofuranoside hydrochloride was added to inhibit the growth of astrocytes. The culture was maintained for three weeks until the time of the experiment.

#### 2.7.2. Electrophysiology

Whole-cell patch-clamp recordings are commonly used to investigate the electrical activity of neurons, including membrane potential (MPs) and action potential (AP) firing. We used an Axon 700 B patch-clamp amplifier (Molecular Devices, Foster City, CA, USA) in conjunction with the Digidata 1440 interface and Clampex/Clampfit software (Version 10, Molecular Devices) to record neuronal activity. The voltage-clamp mode was employed to maintain the membrane potential at −70 mV for whole-cell current recordings. The standard external solution consisted of 150 mM NaCl, 3 mM KCl, 2 mM MgCl2, 2 mM CaCl2, 10 mM HEPES, and 5.5 mM glucose (pH 7.3, adjusted with NaOH; 300–325 mOsm/L). The pipette solution for recording neuronal activity in current-clamp mode contained 140 mM K-gluconate, 7 mM NaCl, 2 mM Mg-ATP, 2 mM Tris-GTP, and 10 mM HEPES (pH 7.3 adjusted with KOH). MPs and AP firing were analyzed using Clampfit 10 software (Molecular Devices).

### 2.8. Statistical Analysis

The data was reported as mean ± SEM, and statistical variances were examined utilizing a one-way ANOVA, succeeded via a Tukey’s test using Prism 8 (GraphPad Software Inc., San Diego, CA, USA). Significance levels were established at *p* < 0.05.

## 3. Results

### 3.1. Quantitative Analysis of CA and KA in KS-126

**Figure 1:**
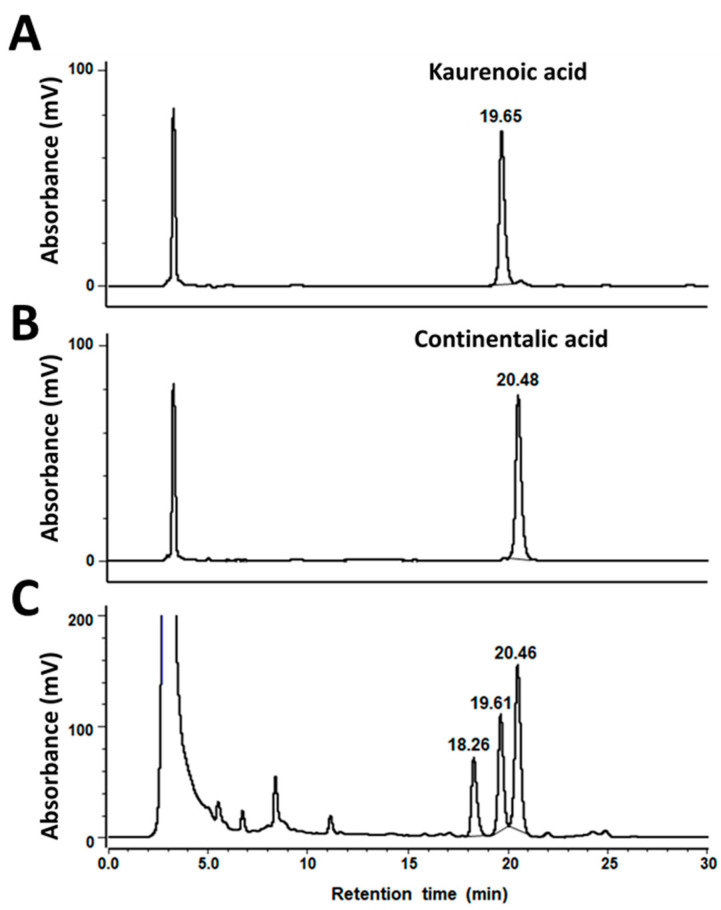
HPLC chromatogram of (**A**) KA and (**B**) CA as a standard compound, and (**C**) *A. continentalis* root extract (KS-126). The concentrations of KA and CA were 28.9 ± 0.8 mg/g and 18.6 ± 0.8 mg/g, respectively.

CA and KA were separated by reverse-phase HPLC using a solvent mixture of acetonitrile in water in the range of 70–80% (*v*/*v*) as the mobile phase. The mobile phase of 80% (*v*/*v*) acetonitrile with water enabled the simultaneous analysis of KA and CA within 25 min (Figure 1). The retention time for the analyses was 19.65 ± 0.07 min for KA and 20.48 ± 0.06 min for CA. Concentrations of KA and CA were 18.6 ± 0.8 mg/g extract and 28.9 ± 0.8 mg/g extract, respectively.

### 3.2. Hypnotic Effects of KS-126

**Figure 2:**
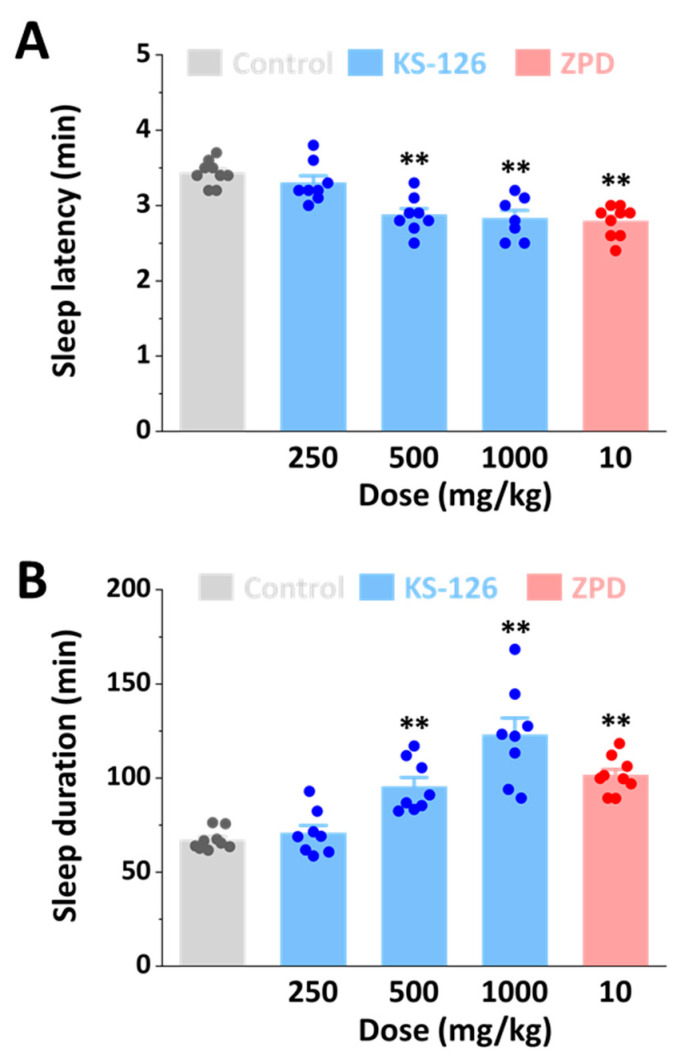
Effects of KS-126 on (**A**) sleep latency and (**B**) sleep duration in pentobarbital-treated ICR mice. Mice were orally administered the vehicle (5% tween 80-saline), KS-126, or ZPD 45 min before the intraperitoneal (i.p.) injection of pentobarbital. The reported values for each group (*n* = 10 per group) represent the mean ± SEM, and the data points are indicated accordingly. ** *p* < 0.01 denotes a significant difference when compared to the control group, as determined via a Tukey’s test.

The hypnotic effects of KS-126 were investigated in ICR mice using a pentobarbital-induced sleep-acceleration test (Figure 2). In the control group, sleep latency and duration were 3.4 ± 0.1 and 67.0 ± 1.8 min, respectively. KS-126 induced a dose-dependent reduction in sleep latency and increase in sleep duration, along with a hypnotic effect observed at a dose of 500 mg/kg of KS-126. When the positive control, ZPD, was administered orally at a dose of 10 mg/kg B.W., it also significantly reduced sleep latency and increased sleep duration in mice treated with a hypnotic dose of pentobarbital.

### 3.3. Effects of KS-126 on Sleep Quantity and Sleep Quality of NREMS in C57BL/6N Mice

**Figure 3:**
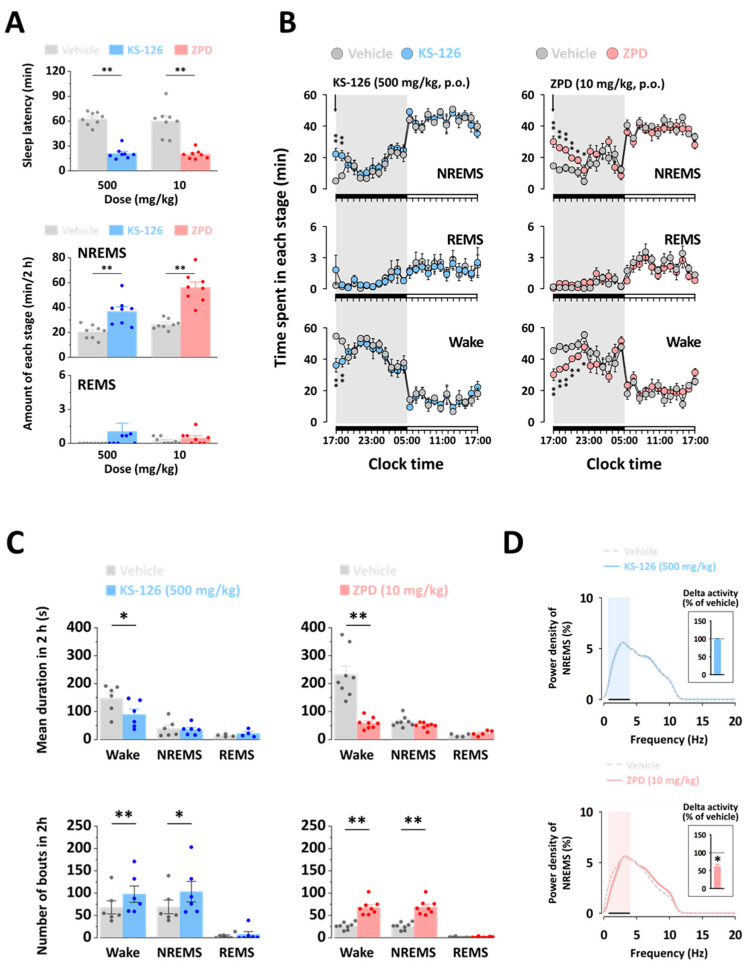
The impacts of KS-126 on sleep quantity and sleep quality of NREMS in C57BL/6N mice. (**A**) Effect of KS-126 on sleep–wake profiles in C57BL/6N mice. Effects of KS-126 on sleep latency. The quantities of NREMS and REMS were assessed during the 2 h following the administration of KS-126. The reported values for each group (*n* = 8 per group) represent the mean ± SEM, and the data points are indicated accordingly. ** *p* < 0.01 denotes a significant difference when compared to each vehicle-treated mice, as determined via a paired Student’s *t*-test. (**B**) Effects of KS-126 on the temporal dynamics of sleep–wake stages for 24 h in C57BL/6N mice. Each circle on the graph represents the hourly mean ± SEM (*n* = 8 per group) of NREMS, REMS, and Wake. * *p* < 0.05 and ** *p* < 0.01 denote significant differences when compared to each vehicle-treated mice, as determined via a paired Student’s *t*-test. Characteristics of sleep–wake episodes (**C**), and the EEG power density of NREMS (**D**) in C57BL/6N mice after oral administration of KS-126. After the administration of KS-126, the mean duration and total number of NREMS, REMS, and Wake bouts were assessed in a 2 h period. The reported values for each group (*n* = 8 per group) represent the mean ± SEM, and the data points are indicated accordingly. * *p* < 0.05 and ** *p* < 0.01 denote significant differences when compared to each vehicle-treated mice, as determined via a paired Student’s *t*-test. The EEG power density of NREMS induced by KS-126: the inset histogram displays the delta activity, which serves as an index of sleep intensity. The dash (–) on the histogram indicates the range of the delta wave (0.5–4 Hz). Significance levels are denoted as * *p* < 0.05 indicating a significant difference when compared to each vehicle-treated mice, as determined via a paired Student’s *t*-test.

We analyzed the sleep structure of C57BL/6N mice using polygraphic recordings to investigate the effects of orally administered KS-126. Values of sleep latency were 21.0 ± 2.4 min in mice treated with 500 mg/kg KS-126, and 19.8 ± 1.9 min in mice treated with 10 mg/kg ZPD (Figure 3A). The sleep latencies for KS-126 and ZPD were 62.9 ± 2.8 min and 60.0 ± 6.3 min, respectively. Both values were significantly shorter compared with the vehicle treatment within each group. Finally, KS-126 induced shorter sleep latency in mice, similar to ZPD, indicating an accelerated onset of NREMS. We calculated the total amounts of NREMS and REMS during a 2 h period following the administration of KS-126 or ZPD (Figure 3A). As expected, the positive control group treated with ZPD showed a significant increase in the total NREMS compared with the vehicle-treated mice (2.1-fold, *p* < 0.01). Similarly, the group administered with KS-126 at 500 mg/kg also significantly increased NREMS (1.8-fold) compared with that in the vehicle control. Furthermore, no significant changes in the amount of REMS were observed following the administration of KS-126 or ZPD compared with their respective vehicle controls.

Figure 3B shows the temporal dynamics of the sleep–wake stage. KS-126 (500 mg/kg) caused a 4.39-fold increase in the amount of NREMS during the first hour and a 2.56-fold increase during the second hour. During the same period, these enhancements were accompanied by a decrease in the Wake period. Following the initial increase in the NREMS, sleep architecture did not show significant changes during the subsequent period. Unlike KS-126, 10 mg/kg ZPD significantly increased the NREMS time by up to 6 h.

To evaluate the sleep-promoting effects of KS-126, we analyzed the mean duration and total number of NREMS, REMS, and waking episodes. KS-126 (500 mg/kg) and ZPD (10 mg/kg) significantly reduced the mean Wake duration by 39.03% and 74.65%, respectively, but did not affect the mean NREMS or REMS durations. Furthermore, KS-126 and ZPD increased the number of NREMS bouts by 1.49-fold and 2.60-fold, respectively, and the number of Wake bouts by 1.43-fold and 2.56-fold, respectively (Figure 3C).

To assess sleep quality, we analyzed the delta activity in the EEG power density during NREMS. As depicted in Figure 3D, there were no observed changes in the EEG power density (0–20 Hz) or delta activity during NREMS between KS-126- and vehicle-treated mice. However, ZPD significantly reduced delta activity compared to that in the vehicle-treated mice. These results suggest that, unlike ZPD, KS-126 increases sleep duration without compromising sleep intensity.

### 3.4. Possible Mechanism of Action for KS-126

**Figure 4:**
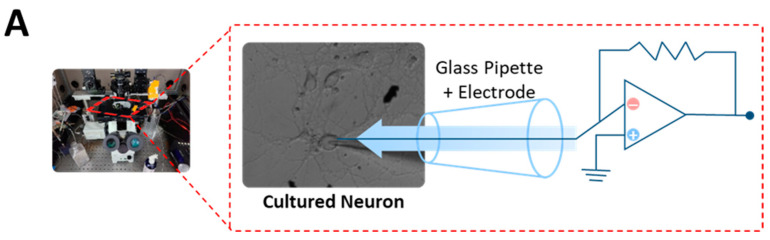

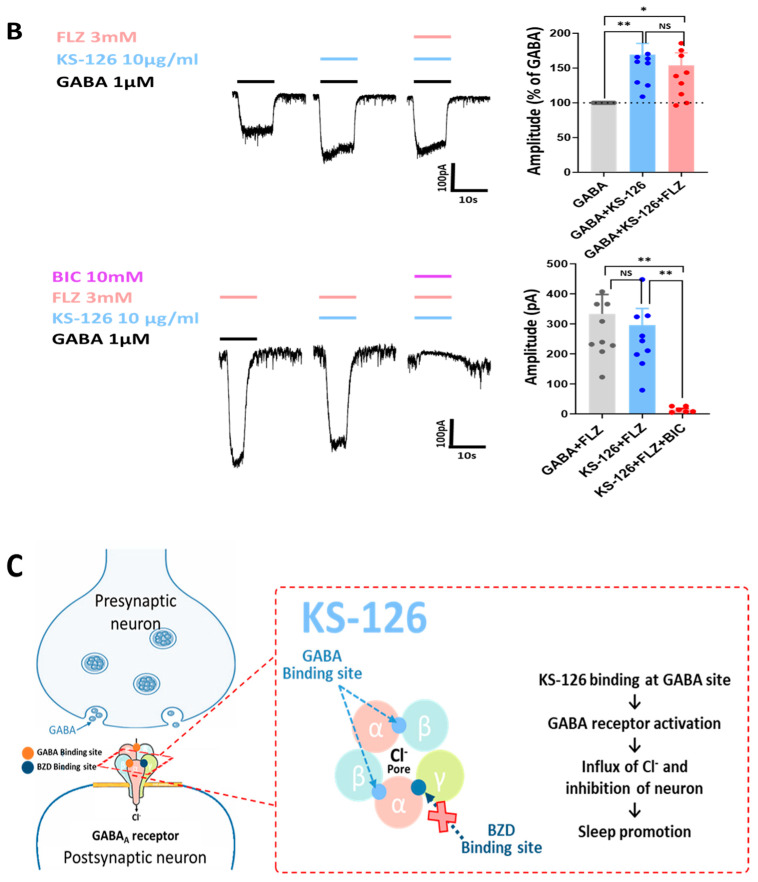
Effect of flumazenil and bicuculline on KS-126 mediated potentiation of GABA currents. (**A**) The schematic diagram illustrates the experimental design used to induce a GABA_A_ receptor-mediated inward current in cultured neurons. (**B**) The left panel shows the representative traces of GABA_A_ receptor-mediated inward currents for GABA alone, GABA + KS-126, GABA + KS-126 + FLZ, and a summary of the mean amplitudes for each treatment normalized by GABA response. The right panel shows the representative traces of GABA_A_ receptor-mediated inward currents for GABA + FLZ, KS-126 + FLZ, KS-126 + FLZ + BIC, and a summary of the amplitudes for each treatment. The reported values for each group (*n* = 6–9 per group) represent the mean ± SEM, and the data points are indicated accordingly. * *p* < 0.05 and ** *p* < 0.01 denote significant differences when compared to the control group, as determined via a Tukey’s test. (**C**) The schematic diagram illustrates our hypothesis regarding the mechanism of action for KS-126-induced sleep promotion.

Figure 4A shows a scheme of the experimental design used to induce GABA_A_ receptor-mediated inward currents in cultured neurons. To investigate the possible interaction between the BDZ-binding site and KS-126, we first evaluated the modulatory effect of KS-126 on GABA currents in the presence of flumazenil, a selective benzodiazepine receptor antagonist. Unexpectedly, the potentiation of the GABA currents by KS-126 (10 μg/mL) was not affected by the presence of 3 μM flumazenil. As a result, our experimental focus shifted to the GABA binding site of the GABA_A_ receptor. However, the GABA currents induced by KS-126 (10 μg/mL) were inhibited when co-treated with the GABA_A_ receptor antagonist bicuculline (10 μM) (Figure 4B). These findings reaffirm that KS-126 promotes sleep through its interaction with the GABA site, not the BDZ-binding site (Figure 4C).

## 4. Discussion

In the current study, we observed that KS-126 reduced sleep latency and increased both sleep duration (Figure 2) and NREMS without affecting REMS (Figure 3). The hypnotic effect of KS-126 was found to be similar to that of ZPD, which was used as a positive control. However, when it comes to sleep quality, mice treated with KS-126 displayed an extended sleep duration without a reduction in sleep intensity, a characteristic that was not observed in mice treated with ZPD (Figure 3). From an electrophysiological perspective, bicuculline, a GABA_A_ receptor antagonist, completely counteracted the hypnotic effect of KS-126 (Figure 4). This implies that KS-126 primarily promotes sleep by positively modulating the GABA_A_ receptors’ GABA binding site rather than the BZD site.

Several natural products, such as herbal medicines, have demonstrated the ability to enhance sleep, suggesting that they may be valuable hypnotic agents [32]. However, their effectiveness and underlying mechanisms, which pose certain limitations, are not fully understood. Therefore, it is crucial to explore the hypnotic potential of these compounds using in vivo animal models. To verify the hypnotic activity of KS-126, we conducted a pentobarbital-induced sleep test in mice, which is a reliable method for evaluating hypnotic properties [33]. As expected, the positive control, ZPD, a non-BZD receptor agonist administered orally at a dose of 10 mg/kg, significantly reduced sleep latency and increased sleep duration in mice, consistent with previous results [34]. In addition, KS-126 dose-dependently decreased sleep latency and increased sleep duration, with hypnotic effects observed at doses of 500 and 1000 mg/kg. The results of our pentobarbital-induced sleep test clearly demonstrated that KS-126 enhanced hypnosis in mice. In addition, our physiological recordings demonstrated that KS-126 led to a significant increase in NREMS during the first 2 h after administration. However, no further alterations in sleep architecture were observed thereafter. These findings indicate that KS-126 triggers NREMS solely during the initial period after administration, with no adverse impacts on subsequent sleep [35]. Considering the noted reduction in average Wake duration and the rise in the count of NREMS episodes caused by KS-126, it is highly likely that KS-126 significantly hampers the ability to sustain wakefulness [35]. In a previous study, delta activity was reported to be an indicator of the depth or intensity of NREMS, which is a measure of sleep quality [36]. Both non-BZD drugs and BZD drugs have been reported to reduce sleep latency and increase sleep duration. However, they have also been reported to suppress delta activity [37,38,39]. ZPD has been reported in several studies to decrease delta activity during NREMS [40,41,42]. Consistent with these reports, our study confirmed that ZPD decreased delta activity. However, KS-126 did not cause any change in delta activity. Our findings suggest that, unlike ZPD, KS-126 increases sleep duration without compromising sleep intensity. This implies that KS-126 induces NREMS, similar to natural physiological sleep [43].

GABA receptors are classified as GABA_A_, GABA_B_, and GABA_C_ receptors [44]. The physiological effects of GABA mostly occur through GABA_A_ receptors, which play a crucial role in functions such as relaxation, sleep, and anesthesia [45,46,47]. BZD enhances the ability of GABA to hyperpolarize cell membranes, thereby facilitating the influx of chloride ions into cells [48]. Consequently, the inhibition of neurotransmission leads to the expression of sedative-hypnotic, anxiolytic, and anticonvulsant effects by these agents [49,50,51]. Therefore, we investigated the effects of KS-126 in the presence of flumazenil, a GABA_A_-BZD receptor antagonist [52], to examine the involvement of specific GABA_A_-BZD receptors in the sleep-promoting effects of KS-126. However, the increase in GABA current induced by KS-126 was unaffected by the presence of flumazenil, and its efficacy was inhibited by bicuculline. According to a previous study, bicuculline is a competitive antagonist of the GABA_A_ receptor that inhibits the binding of GABA to the GABA_A_ receptor [53]. In addition, it blocks calcium-activated potassium channels [54]. As summarized in Figure 4C, KS-126 positively modulates GABA_A_ receptors by interacting with a GABA-binding site [55].

KS-126 and its primary diterpenoids, CA and KA, have been documented to exhibit a range of advantageous effects, including anti-inflammatory, anti-cancer, and wound healing. Particularly, KA has been reported to significantly decrease sleep onset time and increase sleep duration in phenobarbitone-induced sleep test [21]. On the other hand, this study is the first to demonstrate the sleep-promoting effects of KS-126. However, further investigations are essential to explore the efficacy of these diterpenoids in resolving insomnia and elucidate the specific roles of the various active compounds responsible for their sleep-promoting effects. Furthermore, to gain a deeper understanding of the sleep-promoting effects of KS-126, additional investigation is required to explore the sleep efficacy and mechanisms of action of the individual compounds that make up KS-126, both in vivo and in vitro.

## 5. Conclusions

We have shown the sleep-promoting effects of KS-126 and established that these effects are mediated through GABA_A_ receptors, independent of the BZD-binding site. Our findings offer valuable insights that contribute to progress in the field of sedative-hypnotics.

## Data Availability

The data presented in this study are available on request from the corresponding author.
